# Enhanced response inhibition and reduced midfrontal theta activity in experienced Vipassana meditators

**DOI:** 10.1038/s41598-019-49714-9

**Published:** 2019-09-13

**Authors:** Catherine I. Andreu, Ismael Palacios, Cristóbal Moënne-Loccoz, Vladimir López, Ingmar H. A. Franken, Diego Cosmelli, Heleen A. Slagter

**Affiliations:** 1 Escuela de Psicología, Pontificia Universidad Católica de Chile, Santiago, Chile; 2 Millennium Institute for Research in Depression and Personality (MIDAP), Santiago, Chile; 3 Laboratorio de Neurociencia Cognitiva y Social, Facultad de Psicología, Universidad Diego Portales, Santiago, Chile; 4 Department of Health Sciences, Faculty of Medicine, Pontificia Universidad Católica de Chile, Santiago, Chile; 5 Department of Psychology, Education & Child Studies, Erasmus University Rotterdam, Rotterdam, the Netherlands; 6 Department of Psychology, University of Amsterdam, Amsterdam, the Netherlands; 7 Amsterdam Brain and Cognition, University of Amsterdam, Amsterdam, the Netherlands; 8 Department of Experimental and Applied Psychology, Vrije Universiteit Amsterdam, Amsterdam, the Netherlands

**Keywords:** Attention, Cognitive control

## Abstract

Response inhibition - the ability to suppress inappropriate thoughts and actions - is a fundamental aspect of cognitive control. Recent research suggests that mental training by meditation may improve cognitive control. Yet, it is still unclear if and how, at the neural level, long-term meditation practice may affect (emotional) response inhibition. The present study aimed to address this outstanding question, and used an emotional Go/Nogo task and electroencephalography (EEG) to examine possible differences in behavioral and electrophysiological indices of response inhibition between Vipassana meditators and an experience-matched active control group (athletes). Behaviorally, meditators made significantly less errors than controls on the emotional Go/Nogo task, independent of the emotional context, while being equally fast. This improvement in response inhibition at the behavioral level was accompanied by a decrease in midfrontal theta activity in Nogo vs. Go trials in the meditators compared to controls. Yet, no changes in ERP indices of response inhibition, as indexed by the amplitude of the N2 and P3 components, were observed. Finally, the meditators subjectively evaluated the emotional pictures lower in valence and arousal. Collectively, these results suggest that meditation may improve response inhibition and control over emotional reactivity.

## Introduction

The ability to inhibit responses that are inappropriate in a particular context is a core component of cognitive control and critical to successful adaptive behavior^1,2^. Impaired response inhibition is a hallmark of several psychiatric disorders^3^. Over the past two decades, interest in meditation as a method to train and improve cognitive functions, such as response inhibition, has steadily increased. The term ‘meditation’ denotes a wide variety of practices, ranging from concentration techniques to practices that foster wellbeing and altruistic behaviors^4,5^. Many meditation practices also explicitly aim to improve specific cognitive abilities^5^. In line with this, a rapidly growing body of neuroscientific studies shows meditation-related improvements in brain and cognitive function^5–7^.

Accumulating evidence supports the notion that meditation can enhance attention and executive functions^5,8–15^ as well as affective skills^5,16–24^. Recent studies furthermore suggest that meditation may also improve cognitive control. For example, expert meditators show better performance than non-meditators on Stroop interference tasks^12,15,25^ and so do novel practitioners on response inhibition tasks after a meditation retreat^14,26^. Improvements in conflict monitoring as measuring using the Attention Network Test, ANT^27^ have also been found in experienced meditators^28^, and in longitudinal meditation studies^29,30^. Furthermore, reduced emotional interference during cognitive tasks has been described in experienced meditators and in non-meditators after a mindfulness intervention^22,31^. Although the effects of meditation on cognitive control appear quite robust, the neural mechanisms underlying these effects are still unclear.

Few studies have so far examined the effects of meditation on the neural mechanisms underlying cognitive control. Most of these studies used EEG and examined effects of relatively brief meditation interventions on control over automatized reading of words (as measured with the Stroop task) and underlying neural mechanisms, as indexed by the amplitude of the N2 and the frontocental P3 ERP components. The N2, a negative potential that peaks between 200–300 ms after stimulus presentation over midfrontal scalp regions, is associated with top-down inhibitory control and detection of conflict during early, non-motoric stages of inhibition^1,32–34^. The frontocentral P3 is a positive potential with a peak latency between 300–500 ms after stimulus onset. This component has been linked to late-stage inhibition of the motor system itself and outcome evaluation^1,35–40^. While one study reported an increased N2 and reduced P3 conflict effect in participants after practicing 10 min of meditation per day for 5 days per week for 16 weeks compared to a group of waitlist control participants^12^, another study in contrast reported a smaller N2 and an earlier and larger P3 after a 5-hour trial of integrative body-mind training (IBMT), a form of mindfulness meditation, in comparison to a relaxation training control^41^. In yet another study in elderly, a brief meditation intervention was associated with an increased N2 on both trials with and without conflict of the Stroop task^42^. These mixed results may reflect differences between studies in the specific type of meditation practiced, the amount of practice, the age of participants, and/or in how well they controlled for non-specific effects, such as the placebo effect. When participants know that they are participating in a study looking at effects of meditation, this may affect their motivation or expectation to do well, which can hamper the interpretation of results. In longitudinal studies, the incorporation of control groups receiving active interventions (active control group) is therefore essential for excluding possible contributions from such confounding factors^43,44^. Yet, this is equally important in cross-sectional studies, to be able to assign differences in performance between expert meditators and controls to differences in meditation experience per se. Given the broad variety of meditation practices and their differential aims, it is also crucial to specify which type of meditation practice is being investigated and why^4^.

To our knowledge, no study has so far examined effects of long-term meditation experience on the neural dynamics underlying response inhibition with the high temporal resolution of EEG. In the current EEG study, we examined effects of a commonly practiced style of Open Monitoring (OM) meditation, Vipassana^4,45^, on emotional response inhibition, as measured on a Go/Nogo task, while controlling as much as possible for potential non-specific factors by using an active control group consisting of experience-matched athletes with no meditation background. Vipassana meditation is an ancient Buddhist practice that involves cultivating present-moment and non-reactive awareness^45^. Specifically, during this type of meditation, one monitors the content of experience from moment to moment, without evaluation, judgment or affective responding. Therefore, this practice specifically may enhance control over automatic and habitual reactions or response inhibition. The athletes practiced sports for a similar amount of time and period as the meditators. Regular aerobic exercise has also been associated with improvements in cognitive function, including inhibitory control^46–52^. Thereby, the contrast to athletes represents a stringent control of improvements specifically due to the meditative practice. Vipassana meditators and the experience-matched athletes performed an emotional Go/Nogo task while their brain activity was recorded using EEG. Recent studies have shown that response inhibition and emotion are two closely interrelated and mutually dependent processes^53–60^. Increased response inhibition, as reflected by commission errors in Nogo trials, decreased Go reaction times and increased Nogo-P3 amplitudes in positive contexts (compared to neutral and negative), has been reported using an indirect emotional Go/Nogo task, where the Go or Nogo cue was unrelated to the emotional context^54,55^.

Given previous studies showing improved cognitive control in meditators compared to non-meditators, we expected that meditators would display increased response inhibition as evidenced by decreased error rates, particularly in emotional contexts, and by increased N2/P3 amplitudes. We also examined if meditation experience was associated with increased power in the theta range, as previous work suggests that the conflict-related frontocentral EEG signal largely reflects a modulation of ongoing theta-band oscillations during the decision process^61^. As this theta effect is not tightly phase-locked to the stimulus or the response, it may not be captured well by ERPs.

## Results

### Questionnaires

All participants completed a questionnaire concerning demographic data (i.e., age, gender, level of education) and questionnaires regarding how many years of meditation or sports experience they had (depending on the group), and how many hours per week they currently spend practicing them. Also, several questionnaires were used to characterize both groups (described in Methods section). The results from the questionnaires of the complete sample of meditators (N = 31) and controls (N = 30) are shown in Table 1. Although meditators and controls did not significantly differ in total score on the BIS-11 (t (59) = −1.7, p = 0.079), significant differences were found on the cognitive subscale (t (59) = −3.0, p = 0.004), motor subscale (t (59) = −5.8, p < 0.001) and non-planned subscale (t (59) = 2.7, p = 0.009). In general, meditators scored lower on these scales, except for the non-planned subscale that they numerically scored higher. For the PANAS, no difference in self-reported positive affect was found between the two groups (p = 0.8); in contrast, a significant difference in negative affect was found (t (59) = −3.2, p = 0.002), reflecting reduced self-reported negative affect in meditators. Meditators scored significantly higher than controls on four of the FFMQ facets: observe (t (59) = 2.2, p = 0.03), describe (t (59) = 2.7, p = 0.007), non-judge (t (59) = 5.0, p < 0.001) and non-react (t (59) = 4.0, p < 0.001), with awareness being similar between the two groups (t (59) = 1.5, p = 0.1).

**Table 1:** Group scores for the descriptive questionnaires.

	Controls	Meditators	*p*
Total BIS	51.4 (11.9)	46.3 (10.6)	0.079
PANAS Positive	37.0 (7.4)	37.4 (6.6)	0.8
PANAS Negative	21.0 (8.3)	15.1 (5.8)	0.002
FFMQ Observe	30.2 (5.6)	32.9 (3.7)	0.03
FFMQ Describe	27.9 (6.9)	32.2 (5.3)	0.007
FFMQ Awareness	27.8 (6.4)	30.1 (6.0)	0.1
FFMQ Non-judge	22.5 (6.6)	31.1 (6.8)	<0.001
FFMQ Non-react	23.8 (4.2)	28.4 (4.8)	<0.001

Data shown represent means and standard deviations are in parentheses.

**Figure 1:**
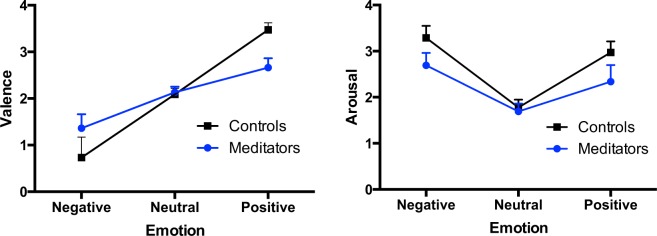
Valence (left) and arousal (right) ratings of the emotional pictures used in the emotional Go/Nogo task. The meditators subjectively evaluated the emotional pictures lower in valence and arousal than the athlete control participants.

In the emotional Go/Nogo task, emotional pictures from were used and then participants rated the pictures in valence and arousal. Table 2 shows the mean and standard deviations on valence and arousal for each type of emotional picture for both groups, plotted in Fig. 1. Regarding valence, as expected, a robust effect of Emotion was found, F(2,110) = 358.09, p < 0.001, with increased valence ratings for positive pictures and decreased valence ratings for negative pictures. No overall difference was found between groups (main Group effect: F(1,55) = 0.48, p = 0.49), but a Group × Emotion interaction was found (F(2,110) = 45.88, p < 0.001). Post-hoc Bonferroni analyzes revealed that differences in valence ratings between groups were significant for negative (t = 6.03, p < 0.001) and positive pictures (t = 7.62, p < 0.001), but not neutral (t = 0.42, p > 0.05). This pattern of results reflected the fact that meditators rated both negative and positive images as lower in valence. As to arousal, as expected, a robust effect of Emotion was found, F(2,110) = 103.6, p < 0.001, with increased arousal ratings for negative and positive pictures compared to neutral. A difference in the arousal assessment was found between groups, with both a main Group (F(1,55) = 27.6, p < 0.001) and Group × Emotion interaction (F(2,110) = 1.32, p = 0.005) effect. Collectively, these effects reflected that meditators self-reported increased mindfulness facets, decreased negative affect and rated emotional pictures lower in valence and arousal.

**Table 2:** Means and standard deviations (in parentheses) of valence (0, negative, to 4, positive) and arousal (0, calming, to 4, arousing) assessments given by the participants for the three types of emotional stimuli.

	Controls	Meditators
Valence Negative	0.73 (0.08)	1.36 (0.08)
Valence Neutral	2.09 (0.05)	2.13 (0.05)
Valence Positive	3.47 (0.09)	2.67 (0.08)
Arousal Negative	3.29 (0.08)	2.69 (0.08)
Arousal Neutral	1.79 (0.09)	1.69 (0.09)
Arousal Positive	2.97 (0.11)	2.34 (0.11)

### Behavioral data

**Figure 2:**
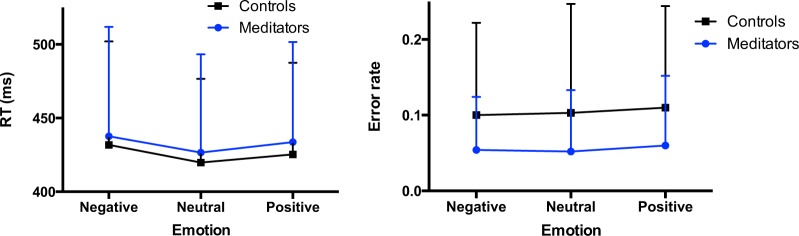
Reaction times for Go trials (left) and total error rates (right) for the emotional Go/Nogo task for each emotional condition. Meditators made significantly fewer errors than controls, while being equally fast on the task.

Reaction times and error rates on the emotional Go/Nogo task are shown in Fig. 2. As expected, more errors were made on the Nogo trials than on the Go (main effect of Inhibition, F (1,58) = 57.99, p < 0.01). Meditators made fewer errors in general compared to controls, as indicated by an overall Group effect, F (1,58) = 7.11, p = 0.01. This effect was not specific to Go or Nogo trials, as no Group × Inhibition interaction was observed (F(1,58) = 2.91, p = 0.093). Error rates were not affected by the emotional valence of the pictures in either group, as no significant effect of emotion was observed (F(2,116) = 2.47, p = 0.095), neither a Group × Emotion interaction effect (F(2,116) = 0.22, p = 0.78). An Inhibition × Emotion interaction effect was observed, F (2,116) = 8.77, p < 0.01, with more errors on Nogo vs. Go trials after positive emotional pictures, compared to neutral and negative. This pattern of findings did not differ between groups, as the Group × Inhibition × Emotion interaction effect was not significant (F(2,116) = 1,26, p = 0.29).

As to reaction times, both in negative and positive emotion Go trials RTs were longer compared to neutral, as indicated by a main effect of Emotion, F (2,116) = 21.12, p < 0.01. No difference between groups was found (F(1,58) = 0.17, p = 0.68) and no Group × Emotion interaction effect was observed (F(2,116) = 0.24, p = 0.79), reflecting no difference in reaction times for the emotional pictures between meditators and controls. Thus, in line with our prediction that Vipassana meditators may show enhanced response inhibition, meditation expertise was selectively associated with reduced error rates on both Go and Nogo trials, but this effect was independent of emotional content.

### Event-related potentials

ERP analyses focused on two well-known markers of response inhibition, the N2 and frontocentral P3.

#### N2

**Figure 3:**
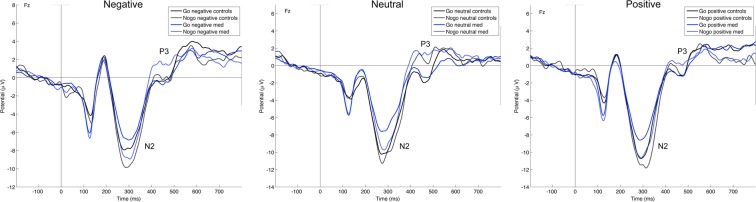
This figure displays grand-average stimulus-locked ERP waveforms at electrode Fz, separately for correct Go and Nogo trials and for negative (left), neutral (central) and positive (right) pictures, for the meditator and control group. Meditation experience was not associated with differences in ERP indices of response inhibition. That is, the difference in N2 and P3 amplitude in NoGo vs. Go trials did not differ between groups.

Figure 3 shows the grand average ERP waveforms separately for meditators and controls at two representative electrodes Fz and Cz. Replicating previous work, the N2 amplitude in Nogo trials was significantly larger than in Go trials, as reflected by a main effect of Inhibition, F(1, 42) = 42.65, p < 0.001. Yet, contrary to our expectation, meditation expertise did not affect the effect of response inhibition on N2 amplitude, as indicated by the absence of a Group × Inhibition interaction (F(1,42) = 1.18, p = 0.28), and a non-significant main effect of Group (F(1,42) = 1.18, p = 0.28). N2 amplitudes were larger for positive pictures, as reflected in a main Emotion effect, F(2,84) = 14.14, p < 0.001. There was true for both groups, as no significant Group × Emotion interaction was observed (F(2,84) = 0.94, p = 0.39), nor a Group × Emotion × Inhibition interaction (F(2,84) = 0.47, p = 0.61). The Emotion × Inhibition interaction was also not significant (F(2,84) = 0.95, p = 0.39). No difference was found between the different electrodes, neither any other interaction effects (all p’s > 0.05), except for a Inhibition × Region interaction (F(3,126) = 3.78, p = 0.017). Thus, meditation experience did not modulate the Go/Nogo N2 effect.

#### P3

As expected and shown in Fig. 3, the P3 amplitude in Nogo trials was also significantly larger than in Go trials, as indicated by a main effect of Inhibition, F(1, 42) = 5.34, p = 0.026. Contrary to our expectation, however, meditation expertise also did not modulate the effect of response inhibition on P3 amplitude, as indicated by a non-significant Group × Inhibition interaction (F(1,42) = 0.85, p = 0.36) and main Group effect (F(1,42) = 0.012, p = 0.91). P3 amplitudes were larger for negative pictures, as captured by a main Emotion effect, F(2,84) = 3.25, p = 0.044. There was no difference between groups for the emotional pictures, as no significant Group × Emotion interaction (F(2,84) = 0.006, p = 0.99), but there was an Emotion × Inhibition interaction (F(2,84) = 5.16, p = 0.009), indicating more positive P3 amplitudes in Nogo vs. Go trials for negative pictures, compared to neutral or positive. No Group × Emotion × Inhibition interaction was observed (F(2,84) = 0.12, p = 0.87). Moreover, the main effect of Region was significant, F(3,126) = 23.57, p < 0.001, indicative of a maximal P3 at electrode Cz. The Region × Inhibition interaction was also significant, F(3,126) = 10.49, p < 0.001. No other significant interaction was found (all p’s > 0.05). Thus, meditation experience did not modulate the effect of response inhibition on the N2 or P3 component. Figs S1 and S2 display the scalp topography of the N2 and P3 ERP components for each condition of interest, separately per group.

### Time- frequency results

**Figure 4:**
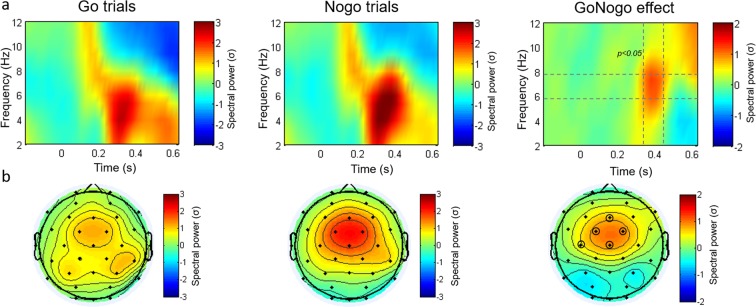
(**a**) Time-frequency plots showing stimulus-locked changes in normalized power in Go trials (left), Nogo trials (center), and in NoGo vs. Go trials (right; the GoNogo effect), across all subjects (meditators and controls) for selected electrodes (Fz, Cz, FC1, FC2 and C3). Grey dotted lines indicate the time-frequency region in which theta power in Nogo trials was significantly higher than in Go trials (permutation test corrected by multiple comparisons). (**b**) Scalp topography maps show the spatial distribution of theta power (6–8 Hz) in Go, Nogo, and Go vs. Nogo trials between 350–450 ms. Electrodes with a black ring denote locations where the GoNogo theta effect was significant. The color scale indicates spectral power in SD.

Successful response inhibition is consistently associated with increased frontal spectral power in the theta band between 200–400 ms in Nogo trials compared to Go trials^1^, that has been shown to be more sensitive to conflict effects than the N2 and P3 ERP components^61^. We therefore next examined the effect of meditation experience on midfrontal theta-band activity (6–8 Hz). To this end, we first examined the presence of this overall GoNogo theta effect in our whole sample, without separating groups. We replicated the GoNogo effect as reflected by a significant increase in frontal theta power in Nogo vs. Go trials between 350–450 ms (Fig. 4. Significant electrodes: Fz, Cz, FC1, FC2 and C3; gray dotted lines indicate the time-frequency region of interests. Permutation test corrected by multiple comparison, p < 0.05).

**Figure 5:**
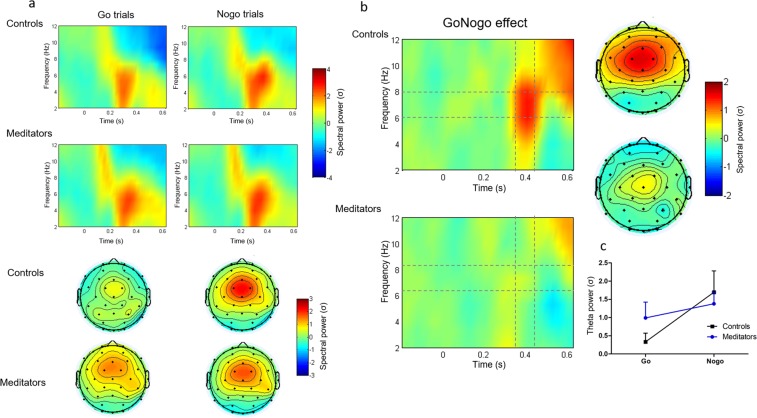
(**a**) Time-frequency charts for the frontocentral electrode cluster (Fz, Cz, FC1, FC2 and C3) and topography of theta power (6–8 Hz) between 350–450 ms, separately for Go and Nogo trials, and for the control and meditator group. (**b**) Time-frequency charts for the frontocentral electrode cluster and scalp topography of GoNogo theta effect (6–8 Hz) between 350–450 ms for the control and meditator group. The GoNogo effect was obtained by subtracting power in Nogo from Go trials. Grey dotted lines indicate the time-frequency regions in which the overall GoNogo theta effect was significant (see Fig. 5a). The color scale indicates spectral power in SD. As can be seen, only the controls displayed the typical increase in theta power in Nogo compared to Go trials. This is further illustrated in (**c**), which shows average theta power (6–8 Hz) between 350–450 ms for the cluster of frontocentral electrodes for Go and Nogo trials, separately for the control and meditator groups.

In order to determine whether the GoNogo theta effect differed between controls and meditators, we compared the GoNogo theta effect between groups using the unbiased ROI (Fig. 4; electrodes: Fz, Cz, FC1, FC2 and C3) and the same time-frequency window for which the overall GoNogo effect was observed (time: 350–450 ms, frequency: 6–8 Hz). We observed an increase in mid-frontal theta activity for both groups and trial types (Fig. 5a). Moreover, replicating previous studies in normal subjects, mid-central theta power was significantly higher in Nogo trials compared with Go trials in the control group (Fig. 5b upper panel, gray dotted lines indicate the time-window of interest. Permutation test corrected by multiple comparisons, p < 0.05). However, the typical GoNogo theta effect observed in the control group was absent in the meditators (Fig. 5b lower panel, gray dotted lines indicate the time-window of interest. Permutation test corrected by multiple comparisons, p < 0.05), who showed a similar pattern of theta activation in Go and Nogo trials (Fig. 5a,c). Importantly, when directly contrasting groups, we found that the GoNogo effect in the control group was significantly greater than the non-significant GoNogo effect in the meditators within the time-window of interest at the selected electrodes (Fig. 5b, gray dotted lines indicate the time-window of interest. Permutation test corrected by multiple comparisons, p < 0.05).

## Discussion

In this EEG study, we examined the effects of a single meditation tradition, Vipassana, on emotional response inhibition and its underlying neural mechanisms. Compared to an experience-matched control group of athletes, Vipassana meditators exhibited lower behavioral omission and commission errors on an emotional Go/Nogo task and rated the emotional pictures lower in valence and arousal. Unexpectedly, we observed no effect of meditation experience on ERP indices of response inhibition (N2 and P3), but meditation experience was associated with decreased midfrontal theta-band power in Nogo trials vs. Go trials. This latter finding corroborates previous evidence suggesting that non-phase locked theta power may be more sensitive to capturing conflict-related midfrontal activity^61^. Importantly, by using an active control group with similar practice time, that was also recruited for their specific expertise, we could better control for potential confounding effects, such as demand characteristics and expectancy effects. Together, the results of our study extend previous findings of enhanced behavioral cognitive control in meditators^14,26^ by showing that experienced meditators in the Vipassana tradition, compared to an experienced-matched control group, exhibit enhanced response inhibition and that changes in midfrontal theta activity may underlie this behavioral benefit.

Behaviorally, meditators made fewer omission and commission errors on the Go/Nogo task, independent of the emotional context provided by the stimuli. This finding is in line with previous reports of improved performance on a non-emotional response inhibition task after a meditation retreat^14,26^ that did not include an active control group. Importantly, while meditators were more accurate on the task, this was not at the expense of response speed, as meditators responded equally fast as controls. Moreover, they displayed a similar emotional interference effect (longer response times in positive and negative compared to neutral contexts). Contrary to our results, other studies have shown reduced emotional interference on cognitive performance in meditators or reduced emotional reactivity in long-term meditators or after a meditation training^16,18,22,24^. This discrepancy in findings may be explained by the nature of our task, an indirect emotional Go/Nogo task, in which emotional content is not explicitly associated with the Go or Nogo stimuli. Despite the absence of group difference in emotional interference during the task, when evaluating the emotional pictures meditators reported decreased arousal levels and provided a more neutral rating of valence. Together, these behavioral and self-report findings indicate that meditation may improve response inhibition and reduce experienced emotional reactivity to pictures. Yet, in a context in which the emotional content of pictures is irrelevant to the task, meditators may be equally affected by their emotional content as athletes.

Given the observed enhanced task performance in meditators, we expected meditation experience to be associated with changes in ERP indices of response inhibition. However, N2/P3 amplitudes in Nogo vs. Go trials did not differ between groups. Previous studies have shown both increased and reduced N2/P3 amplitude effects after meditation training of diverse duration using either a Stroop task or CPT-X task^12,41,42,62^, tasks that are considered to provide a less pure measure of response inhibition than the Go/Nogo task. One possible explanation for the null ERP findings in our study is the use of an active control group (athletes that were matched in duration of practice and recruited for their specific expertise, like the meditators). Most previous meditation studies used control groups of gender/sex/age-matched non-meditators or wait-list control groups in longitudinal studies. However, these control groups control much less well for non-specific factors, such as expectancy effects, than active control groups. Indeed, some studies have shown null results when using active control groups, that undergo some other intervention, such as physical exercise or relaxation^63,64^. Of further importance, athletic training has been shown to modulate the Nogo N2 and P3^50–52^. Another possible explanation for the absence of group differences in the N2/P3 GoNogo effect is thus that meditation and athletic experience modulated these ERP indices of response inhibition to the same extent. Future studies involving a third group of controls without any meditation or athletic training are needed to determine this. Yet, another possible explanation is that meditation practice does not modulate processes specifically reflected in N2 or P3 amplitudes but acts through a different neural mechanism, such as frontal theta oscillations.

Despite the absence of any ERP effects, we found a decrease in midfrontal theta power in Nogo vs. Go trials in meditators compared to controls. This is notable as it has previously been shown that conflict-related midfrontal theta power is non-phased locked, reflective of oscillatory responses, not ERPs^61^. Moreover, that study also found that only midfrontal theta power correlated across-trials with reaction time in a condition-specific (high- vs. low-conflict) manner, while the time-domain phase-locked component did not. These findings suggest that ERPs may not capture midfrontal response inhibition processes as well as theta-band oscillatory activity that is not tightly phase-locked to the stimulus or response. Our findings also suggest that midfrontal theta activity may provide a more sensitive index of response inhibition than the N2 and P3 ERP components, as we selectively observed effects of meditation expertise on midfrontal theta power. This result also highlights the importance of conducting time-frequency analyses of EEG data next to ERP analyses. Midfrontal theta band activity has been well documented as a sensitive marker of conflict detection and action monitoring in different types of situations or tasks^65^. That is, the more difficult and conflictive the situation or task, the more midfrontal theta is typically observed. The observed decreased power in theta in Nogo vs. Go trials may therefore reflect more efficient or decreased conflict detection in the meditators. This would suggest that the observed enhanced performance on the Go/Nogo task in meditators compared to athletes was accomplished by exerting either less or more efficient, rather than enhanced cognitive control per se. Modulations in theta power have been previously related to meditation practice, either to meditation state, resting state or during a cognitive task^66–69^. Interestingly, in our study, meditators made fewer errors on both Go and Nogo trials. It is likely that meditators performed the task with an equanimous approach^70^, and therefore were less reactive in any type of trial^71^. This could also explain their similar levels of mid-frontal theta power in Go and Nogo trials. In addition, it is also possible that they showed enhanced sustained attention or performance monitoring during the task^72,73^, enabling faster online error correction.

The present study used a cross-sectional design, preventing drawing conclusions on causality. Longitudinal designs measuring emotional response inhibition before and after meditations interventions compared to an active control intervention are needed to confirm that the observed behavioral and neural effects are related to meditation experience per se, rather than preexisting differences between the meditators and controls. Future longitudinal work should also determine the amount and duration of meditation practice required to induce behavioral and/or neural changes^14,31^. Also, since the controls in our study were expert athletes, we cannot fully exclude the possibility that (some of) the observed effects are not (at least in part) related to athletic experience. Yet, as many studies in healthy adults have shown conflict-related midfrontal theta activity^61^, and this effect was lacking in the meditators, but present in the athletes. This supports an interpretation of our findings in terms of an effect of meditation expertise on response inhibition.

To conclude, compared to athletes, Vipassana meditators displayed enhanced response inhibition at the behavioral level, while being equally fast, and this behavioral effect was accompanied by reduced inhibition-related midfrontal theta activity. No differences were found between meditators and controls in GoNogo N2/P3 amplitudes. Together, our results suggest that meditation may improve response inhibition and cognitive control, and also highlight the importance of including active control groups.

## Methods

Next to an emotional GoNoGo task, the meditators and controls performed an Eriksen Flanker task, the results of which have been reported elsewhere^74^. The description of the Methods below is thus based in part on the methods description in our previous report.

### Participants

Thirty-one Vipassana meditators and thirty non-meditator athletes (i.e., controls) participated in the study. In order to participate in the study, participants had to currently practice either Vipassana meditation or a sport at least 3 times a week, one hour each time, for at least one year. Both groups of participants were recruited separately via announcements in meditation or sport centers, respectively, stating the possibility to participate in an experiment to assess the effects of meditation or sport practice on brain function. Participants in one group (experimental or control) did not know about the existence of the other group until after the experiment. Exclusion criteria for both groups included current self-reported neurological or psychiatric illness. All meditators reported at least 1 year of exclusively Vipassana meditation practice (M = 5.1 years, SD = 3.73) with total 2500 mean hours of meditation (range 375–12550; s.d. = 2658). The control group consisted of athletes of different kinds of practices with no prior experience with meditation. All athletes reported at least one year of regular exercise (M = 7.1 years, s.d. = 5.62) with total 2460 mean hours of exercise (range 144–11520; s.d. = 2492). The mean number of hours of practice did not differ between groups, t (59) = 0.063, p = 0.95; neither did the mean years of practice, t (59) = 1.69, p = 0.09. The mean age (t (59) = 1.11, p = 0.26) and gender ratio (chi-square = 0.02, p = 0.89) of both groups did not differ. For our behavioral analyses, we eliminated one participant due to equipment malfunction. Sixteen additional participants were excluded from the EEG analyses due to too few artifact-free EEG epochs (less than 20) to calculate a reliable N2 and P3^75^. This left a total of 24 meditators and 20 controls for the EEG analyses. The mean number of hours of practice of the EEG subsample did not differ between groups, t (42) = 0.69, p = 0.49; neither the mean years of practice, t (42) = 0.79, p = 0.43. The mean age (t (42) = 0.73, p = 0.46) and gender ratio (chi-square = 0.20, p = 0.65) of both groups in the EEG subsample did not differ. The ethics committee of the Pontificia Universidad Católica de Chile approved the study. All procedures were carried out with the adequate understanding of the participants and were done in accordance with the ethics committee and Helsinski declaration standards. Informed consent was obtained from all participants.

### Questionnaires

In addition to answers to several demographic and meditation/sports experience questions, several questionnaires were used to characterize both groups of participants. The Five Facet Mindfulness Questionnaire, FFMQ^76^, measures five factors that represent subcomponents of mindfulness: observing, describing, acting with awareness, non-judging of inner experience, and non-reactivity to inner experience. Positive and negative affect were measured by the Positive and Negative Affect Scales, PANAS^77^. The Barratt Impulsiveness Scale, BIS-11^78^ was used to measure trait impulsivity.

### Task and procedure

Upon arrival, participants first signed an informed consent and completed all questionnaires. Participants were next seated in a comfortable EEG chair in a light- and sound-attenuated room. Electrodes were placed and task instructions were provided, after which the modified emotional Go/Nogo task^54^ was performed while EEG and behavioral data were recorded. 50 negative, neutral and positive pictures were presented, all selected from the EmoMadrid affective picture database^79^, http://www.uam.es/CEACO/EmoMadrid.htm. Each picture was presented for 300 ms and had a blue or purple frame. Frame color indicated whether a stimulus was a Go or a Nogo stimulus. After the stimulus, a black screen was presented with a white central fixation-cross for a duration that randomly varied between 800 ms and 1200 ms (Fig. 1). Participants were asked to respond to stimuli in Go trials by pressing a button as fast as possible and to withhold their response in Nogo trials. They were explicitly instructed to maintain high accuracy during the whole task. A high percentage of Go cues (66.67%) were employed to increase the tendency to respond. The order of trial type (Go versus Nogo) was quasi randomized such that at most four Go and two Nogo trials were presented consecutively. Participants completed the emotional Go/Nogo task in four blocks of 90 trials each and could take a short break between blocks. Each block involved 30 pictures (10 neutral, 10 positive and 10 negative), each one presented 3 times (two as Go and one as Nogo). In this way, the emotional Go/Nogo task consisted of 360 trials (240 Go and 120 Nogo trials). Before the start of the task, participants performed 12 practice trials, involving additional neutral pictures. Total task duration was about 10 minutes. At the end of the experiment, each participant filled out a bidimensional-scaling test of each picture, assessing its valence and arousal level with a Likert scale. After the emotional Go/Nogo experiment participants performed an Eriksen-Flanker task, the results of which have been reported previously^74^.

### EEG recording and processing

EEG signals were recorded using a Biosemi Active-Two amplifier system and 32 scalp Ag/AgCl electrodes mounted in an elastic cap according to the 10–20 system. Six additional electrodes were fixed on the left and right mastoids, the two outer canthi of both eyes (HEOG), and below and above the left eye (VEOG). All signals were digitized with a sampling rate of 2048 Hz and 24-bit A/D conversion. Data were off-line re-referenced to average mastoids. Off-line, EEG and EOG activity was bandpass filtered between 0.05–35 Hz (phase shift-free Butterworth filter; 24 dB/octave slope). For the ERP analyses, data were segmented in epochs of 1 s (200 ms before and 800 ms after stimulus onset). After ocular correction, trials in which the EEG signal exceeded ±100 mV were automatically excluded from the analyses. After baseline correction (−200 to 0 ms), artifact-free trials were averaged to obtain ERPs at each scalp site separately for the six conditions of interest (all correct Go neutral, Go negative, Go positive, Nogo neutral, Nogo negative, Nogo positive trials). The N2 was defined as the mean value in the 250–350 ms time interval after stimulus onset at a cluster of frontocentral electrodes, including Fz, FC1, FC2 and Cz^80^. The P3 was defined as the mean value in the 400–500 ms time window after stimulus onset at a cluster of central electrodes including Fz, Cz, C3 and C4^80^. The mean number of analyzable Go and Nogo epochs was 96.5 and 46.3 for neutral pictures, 95.6 and 48.6 for negative pictures and 95.1 and 46.7 for positive pictures, respectively. The number of trials did not differ between groups in any condition (all p’s > 0.05).

Time-frequency analysis was performed using the Matlab FieldTrip toolbox^81^. Filtered data between 0.05–35 Hz (phase shift-free Butterworth filter; 24 dB/octave slope) was segmented between [−0.5, 1] seconds around stimulus onset separately for Go and Nogo trials for each group (given the behavioral and ERP results, the time-frequency analysis did not include the different emotional contexts). Total power spectrum was obtained by transforming each epoch into the frequency domain using a sequential and overlapping unique Hanning window of 250 ms in steps of 25 ms with the multitaper time-frequency transformation (MTMCONVOL from ft_freqanalysis Fieldtrip software) method. Additionally, the convolution function includes a ‘Zero’ type padding designed to solve border effects. After the transformation, we obtained a time-frequency spectrum with 1 Hz and 250 ms resolution. Finally, the results were Z-normalized, taking as baseline the 350 ms before the stimulus onset.

### Statistical analysis

Repeated measurement ANOVAs with Greenhouse–Geisser adjusted p-values were used with Group (meditators and controls) in all analyses as a between-subjects factor. For the assessments of differences in self-reported valence and arousal, two 2 × 3 ANOVA Group × Emotion (negative, neutral and positive pictures) were computed. To examine effects of expertise on behavioral error rate, we employed a 2 × 2 × 3 repeated measures ANOVA: Group × Inhibition (errors on go trials vs. Nogo trials) × Emotion (negative, neutral and positive trials). For the behavioral reaction time data, we employed a 2 × 3 repeated measures ANOVA: Group × Emotion RT (RTs on correct Go negative, neutral and positive trials). To examine effects of meditation expertise on ERP indices of response inhibition (i.e., the N2 and P3), we conducted repeated measures ANOVA, with Group as between subjects factor, and Inhibition (Go and Nogo trials), Emotion (negative, neutral and positive) and Region (electrodes described above for each ERP component) as within subject factors. For all analyses, the level of significance employed was 0.05.

The time frequency analysis focused on examining differences in frontal theta (6–8 Hz) activity between Go and NoGo trials between groups. To this end, first, a permutation test was performed for each time bin of interest (350–450 ms), including a correction for multiple comparison^82–84^ to identify the latency and scalp topography of the overall GoNogo theta effect, without separating groups. Under the null hypothesis of no differences, the different conditions (Go and Nogo trials) are drawn from the same single distribution. Thereby, two random sets of trials from the complete sample can be chosen without expecting any “condition” differences. The first step of the permutation test was therefore to randomly choose two sets of trials from the complete sample of (Go and Nogo) trials and calculate a *t-test* for each time bin. This step was repeated 1000 times and the highest *t*-value of each permutation was included in the permutation distribution to correct for multiple comparisons^82^. Finally, from the thus obtained distribution under the null hypothesis, the 5^th^ percentile threshold value was used to determine the statistical significance of *t*-values obtained by statistically comparing theta power between Go and NoGo trials across all participants. All values above this threshold were considered statistically significant (p < 0.05). This statistical procedure was performed initially for each electrode and all participants (20 per group; selection based on the number of trials per condition), allowing us to find the specific electrodes that replicate the previously described GoNogo theta effect. The final statistics and figures include the average of only the selected electrodes. In order to find whether the GoNogo effect was present in both groups, the procedure was repeated separately for the controls and meditators participants, using the average of the selected electrodes for the theta window, for each time window of interest. Finally, differences in the GoNogo effect between groups were assessed using the same procedure and parameters mentioned above. It is important to note that here the theta ROI was defined based on the overall contrast, orthogonal to the group effect.

## Supplementary information

Supplementary Figures

## Data Availability

The datasets generated during and/or analyzed during the current study are available from the corresponding author on reasonable request.

## References

[1] Huster RJ, Enriquez-Geppert S, Lavallee CF, Falkenstein M, Herrmann CS (2013). Electroencephalography of response inhibition tasks: functional networks and cognitive contributions. Int J Psychophysiol.

[2] Albert J, Lopez-Martin S, Hinojosa JA, Carretie L (2013). Spatiotemporal characterization of response inhibition. Neuroimage.

[3] Chambers CD, Garavan H, Bellgrove MA (2009). Insights into the neural basis of response inhibition from cognitive and clinical neuroscience. Neuroscience and biobehavioral reviews.

[4] Slagter HA, Davidson RJ, Lutz A (2011). Mental training as a tool in the neuroscientific study of brain and cognitive plasticity. Frontiers in human neuroscience.

[5] Tang YY, Holzel BK, Posner MI (2015). The neuroscience of mindfulness meditation. Nature Reviews Neuroscience.

[6] Cahn BR, Polich J (2006). Meditation states and traits: EEG, ERP, and neuroimaging studies. Psychological bulletin.

[7] Chiesa A, Calati R, Serretti A (2011). Does mindfulness training improve cognitive abilities? A systematic review of neuropsychological findings. Clinical psychology review.

[8] Lutz A, Slagter HA, Dunne JD, Davidson RJ (2008). Attention regulation and monitoring in meditation. Trends in cognitive sciences.

[9] Brefczynski-Lewis JA, Lutz A, Schaefer HS, Levinson DB, Davidson RJ (2007). Neural correlates of attentional expertise in long-term meditation practitioners. Proceedings of the National Academy of Sciences of the United States of America.

[10] Hasenkamp W, Barsalou LW (2012). Effects of meditation experience on functional connectivity of distributed brain networks. Frontiers in human neuroscience.

[11] Lutz A (2009). Mental training enhances attentional stability: neural and behavioral evidence. The Journal of Neuroscience.

[12] Moore A, Gruber T, Derose J, Malinowski P (2012). Regular, brief mindfulness meditation practice improves electrophysiological markers of attentional control. Frontiers in human neuroscience.

[13] Slagter HA (2007). Mental training affects distribution of limited brain resources. PLoS biology.

[14] Zanesco AP, King BG, Maclean KA, Saron CD (2013). Executive control and felt concentrative engagement following intensive meditation training. Frontiers in human neuroscience.

[15] Moore A, Malinowski P (2009). Meditation, mindfulness and cognitive flexibility. Consciousness and cognition.

[16] Brown KW, Goodman RJ, Inzlicht M (2013). Dispositional mindfulness and the attenuation of neural responses to emotional stimuli. Social Cognitive Affective Neuroscience.

[17] Chambers R, Gullone E, Allen NB (2009). Mindful emotion regulation: An integrative review. Clinical psychology review.

[18] Desbordes G (2012). Effects of mindful-attention and compassion meditation training on amygdala response to emotional stimuli in an ordinary, non-meditative state. Frontiers in human neuroscience.

[19] Farb NA, Anderson AK, Segal ZV (2012). The mindful brain and emotion regulation in mood disorders. Canadian Journal of Psychiatry.

[20] Klimecki OM, Leiberg S, Lamm C, Singer T (2013). Functional neural plasticity and associated changes in positive affect after compassion training. Cerebral Cortex.

[21] Lutz A, Brefczynski-Lewis J, Johnstone T, Davidson RJ (2008). Regulation of the neural circuitry of emotion by compassion meditation: effects of meditative expertise. PLoS One.

[22] Ortner C, Kilner S, Zelazo PD (2007). Mindfulness meditation and reduced emotional interference on a cognitive task. Motivation and Emotion.

[23] Paul NA, Stanton SJ, Greeson JM, Smoski MJ, Wang L (2013). Psychological and neural mechanisms of trait mindfulness in reducing depression vulnerability. Social Cognitive Affective Neuroscience.

[24] Taylor VA (2011). Impact of mindfulness on the neural responses to emotional pictures in experienced and beginner meditators. Neuroimage.

[25] Teper R, Inzlicht M (2013). Meditation, mindfulness and executive control: the importance of emotional acceptance and brain-based performance monitoring. Social Cognitive Affective Neuroscience.

[26] Sahdra BK (2011). Enhanced response inhibition during intensive meditation training predicts improvements in self-reported adaptive socioemotional functioning. Emotion.

[27] Fan J, McCandliss BD, Sommer T, Raz A, Posner MI (2002). Testing the efficiency and independence of attentional networks. Journal of cognitive neuroscience.

[28] Jha AP, Krompinger J, Baime MJ (2007). Mindfulness training modifies subsystems of attention. Cognitive, Affective and Behavioral Neuroscience.

[29] Elliott JC, Wallace BA, Giesbrecht B (2014). A week-long meditation retreat decouples behavioral measures of the alerting and executive attention networks. Frontiers in human neuroscience.

[30] Tang YY (2007). Short-term meditation training improves attention and self-regulation. Proceedings of the National Academy of Sciences of the United States of America.

[31] Allen M (2012). Cognitive-affective neural plasticity following active-controlled mindfulness intervention. Journal of Neuroscience.

[32] Nieuwenhuis S, Yeung N, van den Wildenberg W, Ridderinkhof KR (2003). Electrophysiological correlates of anterior cingulate function in a go/no-go task: effects of response conflict and trial type frequency. Cognitive, affective & behavioral neuroscience.

[33] Falkenstein M (2006). Inhibition, conflict and the Nogo-N2. Clinical neurophysiology: official journal of the International Federation of Clinical Neurophysiology.

[34] Kaiser S (2006). N2 event-related potential correlates of response inhibition in an auditory Go/Nogo task. Int J Psychophysiol.

[35] Dimoska A, Johnstone SJ, Barry RJ (2006). The auditory-evoked N2 and P3 components in the stop-signal task: indices of inhibition, response-conflict or error-detection?. Brain and cognition.

[36] Smith JL, Jamadar S, Provost AL, Michie PT (2013). Motor and non-motor inhibition in the Go/NoGo task: an ERP and fMRI study. Int J Psychophysiol.

[37] Smith JL, Johnstone SJ, Barry RJ (2008). Movement-related potentials in the Go/NoGo task: the P3 reflects both cognitive and motor inhibition. Clinical neurophysiology: official journal of the International Federation of Clinical Neurophysiology.

[38] Band GP, van Boxtel GJ (1999). Inhibitory motor control in stop paradigms: review and reinterpretation of neural mechanisms. Acta Psychol (Amst).

[39] Wessel JR, Aron AR (2015). It’s not too late: the onset of the frontocentral P3 indexes successful response inhibition in the stop-signal paradigm. Psychophysiology.

[40] Waller DA, Hazeltine E, Wessel JR (2019). Common neural processes during action-stopping and infrequent stimulus detection: The frontocentral P3 as an index of generic motor inhibition. Int J Psychophysiol.

[41] Fan Y, Tang YY, Tang R, Posner MI (2015). Time course of conflict processing modulated by brief meditation training. Front Psychol.

[42] Malinowski Peter, Moore Adam W., Mead Bethan R., Gruber Thomas (2015). Mindful Aging: The Effects of Regular Brief Mindfulness Practice on Electrophysiological Markers of Cognitive and Affective Processing in Older Adults. Mindfulness.

[43] Davidson RJ (2010). Empirical explorations of mindfulness: conceptual and methodological conundrums. Emotion.

[44] Tang YY, Posner MI (2013). Tools of the trade: theory and method in mindfulness neuroscience. Social Cognitive Affective Neuroscience.

[45] Hart, W. The Art of Living: Vipassana Meditation as Taught by S. N. Goenka. *New York, NY: HarperOne* (1987).

[46] Kida N, Oda S, Matsumura M (2005). Intensive baseball practice improves the Go/Nogo reaction time, but not the simple reaction time. Brain Research Cognitive Brain Research.

[47] Zhao Emily, Tranovich Michael J., DeAngelo Ron, Kontos Anthony P., Wright Vonda J. (2015). Chronic exercise preserves brain function in masters athletes when compared to sedentary counterparts. The Physician and Sportsmedicine.

[48] Peiffer R, Darby LA, Fullenkamp A, Morgan AL (2015). Effects of Acute Aerobic Exercise on Executive Function in Older Women. Journal of Sports Science and Medicine.

[49] Eggenberger P, Theill N, Holenstein S, Schumacher V, de Bruin ED (2015). Multicomponent physical exercise with simultaneous cognitive training to enhance dual-task walking of older adults: a secondary analysis of a 6-month randomized controlled trial with 1-year follow-up. Clinical interventions in aging.

[50] Endo H, Kato Y, Kizuka T, Takeda T (2006). A comparison of stimulus synchronous activity in the primary motor cortices of athletes and non-athletes. Exp Brain Res.

[51] Yamashiro K (2015). Skill-Specific Changes in Somatosensory Nogo Potentials in Baseball Players. PLoS One.

[52] Nakamoto H, Mori S (2008). Effects of stimulus-response compatibility in mediating expert performance in baseball players. Brain research.

[53] Brown MR (2012). Effects of emotional context on impulse control. Neuroimage.

[54] Albert J, Lopez-Martin S, Tapia M, Montoya D, Carretie L (2012). The role of the anterior cingulate cortex in emotional response inhibition. Human brain mapping.

[55] Albert J, Lopez-Martin S, Carretie L (2010). Emotional context modulates response inhibition: neural and behavioral data. Neuroimage.

[56] Schulz KP (2007). Does the emotional go/no-go task really measure behavioral inhibition? Convergence with measures on a non-emotional analog. Archives of clinical neuropsychology: the official journal of the National Academy of Neuropsychologists.

[57] Zhang W, Lu J (2012). Time course of automatic emotion regulation during a facial Go/Nogo task. Biological psychology.

[58] Goldstein M (2007). Neural substrates of the interaction of emotional stimulus processing and motor inhibitory control: an emotional linguistic go/no-go fMRI study. Neuroimage.

[59] Schulz KP (2009). Dissociable neural effects of stimulus valence and preceding context during the inhibition of responses to emotional faces. Human brain mapping.

[60] Shafritz KM, Collins SH, Blumberg HP (2006). The interaction of emotional and cognitive neural systems in emotionally guided response inhibition. Neuroimage.

[61] Cohen MX, Donner TH (2013). Midfrontal conflict-related theta-band power reflects neural oscillations that predict behavior. J Neurophysiol.

[62] Schoenberg PL (2014). Effects of mindfulness-based cognitive therapy on neurophysiological correlates of performance monitoring in adult attention-deficit/hyperactivity disorder. Clinical neurophysiology: official journal of the International Federation of Clinical Neurophysiology.

[63] MacCoon DG, MacLean KA, Davidson RJ, Saron CD, Lutz A (2014). No sustained attention differences in a longitudinal randomized trial comparing mindfulness based stress reduction versus active control. PLoS One.

[64] Farias Miguel, Wikholm Catherine (2016). Has the science of mindfulness lost its mind?. BJPsych Bulletin.

[65] Nigbur R, Ivanova G, Stürmer B (2011). Theta power as a marker for cognitive interference. Clinical Neurophysiology.

[66] Lomas T, Ivtzan I, Fu CH (2015). A systematic review of the neurophysiology of mindfulness on EEG oscillations. Neuroscience and biobehavioral reviews.

[67] Slagter HA, Lutz A, Greischar LL, Nieuwenhuis S, Davidson RJ (2009). Theta phase synchrony and conscious target perception: impact of intensive mental training. J Cogn Neurosci.

[68] Tang YY (2009). Central and autonomic nervous system interaction is altered by short-term meditation. Proceedings of the National Academy of Sciences of the United States of America.

[69] Jo HG, Malinowski P, Schmidt S (2017). Frontal Theta Dynamics during Response Conflict in Long-Term Mindfulness Meditators. Front Hum Neurosci.

[70] Desbordes Gaëlle, Gard Tim, Hoge Elizabeth A., Hölzel Britta K., Kerr Catherine, Lazar Sara W., Olendzki Andrew, Vago David R. (2014). Moving Beyond Mindfulness: Defining Equanimity as an Outcome Measure in Meditation and Contemplative Research. Mindfulness.

[71] Baquedano C (2017). Compared to self-immersion, mindful attention reduces salivation and automatic food bias. Scientific reports.

[72] Lutz A, Greischar LL, Rawlings NB, Ricard M, Davidson RJ (2004). Long-term meditators self-induce high-amplitude gamma synchrony during mental practice. Proceedings of the national Academy of Sciences.

[73] Basso JC, McHale A, Ende V, Oberlin DJ, Suzuki WA (2019). Brief, daily meditation enhances attention, memory, mood, and emotional regulation in non-experienced meditators. Behavioural brain research.

[74] Andreu CI (2017). Behavioral and Electrophysiological Evidence of Enhanced Performance Monitoring in Meditators. Mindfulness.

[75] Rietdijk WJ, Franken IH, Thurik AR (2014). Internal consistency of event-related potentials associated with cognitive control: N2/P3 and ERN/Pe. PLoS One.

[76] Baer RA, Smith GT, Hopkins J, Krietemeyer J, Toney L (2006). Using self-report assessment methods to explore facets of mindfulness. Assessment.

[77] Watson D, Clark LA, Tellegen A (1988). Development and validation of brief measures of positive and negative affect: the PANAS scales. Journal of personality and social psychology.

[78] Patton JH, Stanford MS, Barratt ES (1995). Factor structure of the Barratt impulsiveness scale. Journal of clinical psychology.

[79] Carretié, L., Tapia, M., López-Martín, S. & Albert, J. EmoMadrid: An emotional pictures database for affect research. *Motivation and Emotion*, 10.1007/s11031-019-09780-y (2019).

[80] Luijten M, Littel M, Franken IH (2011). Deficits in inhibitory control in smokers during a Go/NoGo task: an investigation using event-related brain potentials. PLoS One.

[81] Oostenveld R, Fries P, Maris E, Schoffelen JM (2011). FieldTrip: Open source software for advanced analysis of MEG, EEG, and invasive electrophysiological data. Comput Intell Neurosci.

[82] Bosman CA (2012). Attentional stimulus selection through selective synchronization between monkey visual areas. Neuron.

[83] Maris E, Oostenveld R (2007). Nonparametric statistical testing of EEG-and MEG-data. Journal of neuroscience methods.

[84] Nichols TE, Holmes AP (2002). Nonparametric permutation tests for functional neuroimaging: a primer with examples. Human brain mapping.

