# Supplementary material for: Enhanced response inhibition and reduced midfrontal theta activity in experienced Vipassana meditators
Source: Sci Rep. 2019 Sep 13;9:13215. doi: 10.1038/s41598-019-49714-9 (PMC6744491; doi:10.1038/s41598-019-49714-9)
Supplement: Supplementary file 1 — Supplementary Figures [file 41598_2019_49714_MOESM1_ESM.pdf]

Enhanced response inhibition and reduced midfrontal theta activity in experienced Vipassana meditators

Catherine I. Andreu[1,2*], Ismael Palacios[1,3], Cristóbal Moënne-Loccoz[4], Vladimir López[1], Ingmar H.A. Franken[5], Diego Cosmelli[1, 2], Heleen A. Slagter[6,7,8]

[1] Escuela de Psicología, Pontificia Universidad Católica de Chile, Chile

[2] Millennium Institute for Research in Depression and Personality (MIDAP), Chile

[3] Laboratorio de Neurociencia Cognitiva y Social, Facultad de Psicología, Universidad Diego Portales, Chile

[4] Biomedical Neuroscience Institute, Facultad de Medicina, Universidad de Chile, Santiago, Chile

[5] Department of Psychology, Education & Child Studies, Erasmus University Rotterdam, the Netherlands

[6] Department of Psychology, University of Amsterdam, the Netherlands

[7] Amsterdam Brain and Cognition, University of Amsterdam, the Netherlands

[8] Department of Experimental and Applied Psychology, Vrije Universiteit Amsterdam, the Netherlands

*** Corresponding Author:**

E-mail: ciandreu@uc.cl (CIA)

SUPPLEMENTARY FIGURE LEGENDS:

Fig S1. Scalp voltage maps show the spatial distribution of grand-average ERP amplitude within 250–350 ms (N2 time window), separately for correct Go and Nogo trials for negative (left), neutral (central) and positive (right) pictures, for controls and meditators.

Fig S2. Scalp voltage maps show the spatial distribution of grand-average ERP amplitude within 400–500 ms (P3 time window), separately for correct Go and Nogo trials for negative (left), neutral (central) and positive (right) pictures, for controls and meditators.

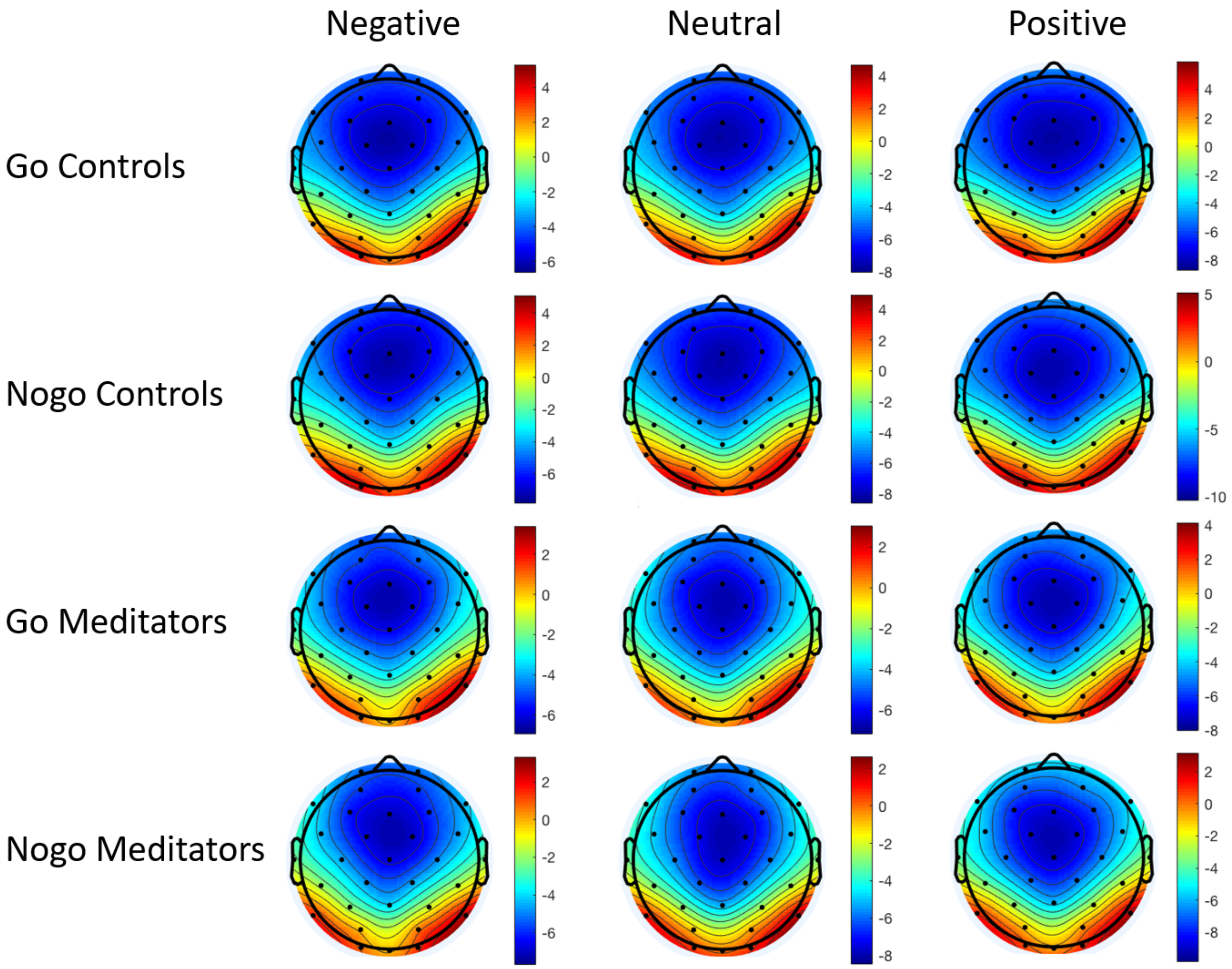
Negative
Neutral
Positive
Go Controls
Nogo Controls
Go Meditators
Nogo Meditators

Fig S1.

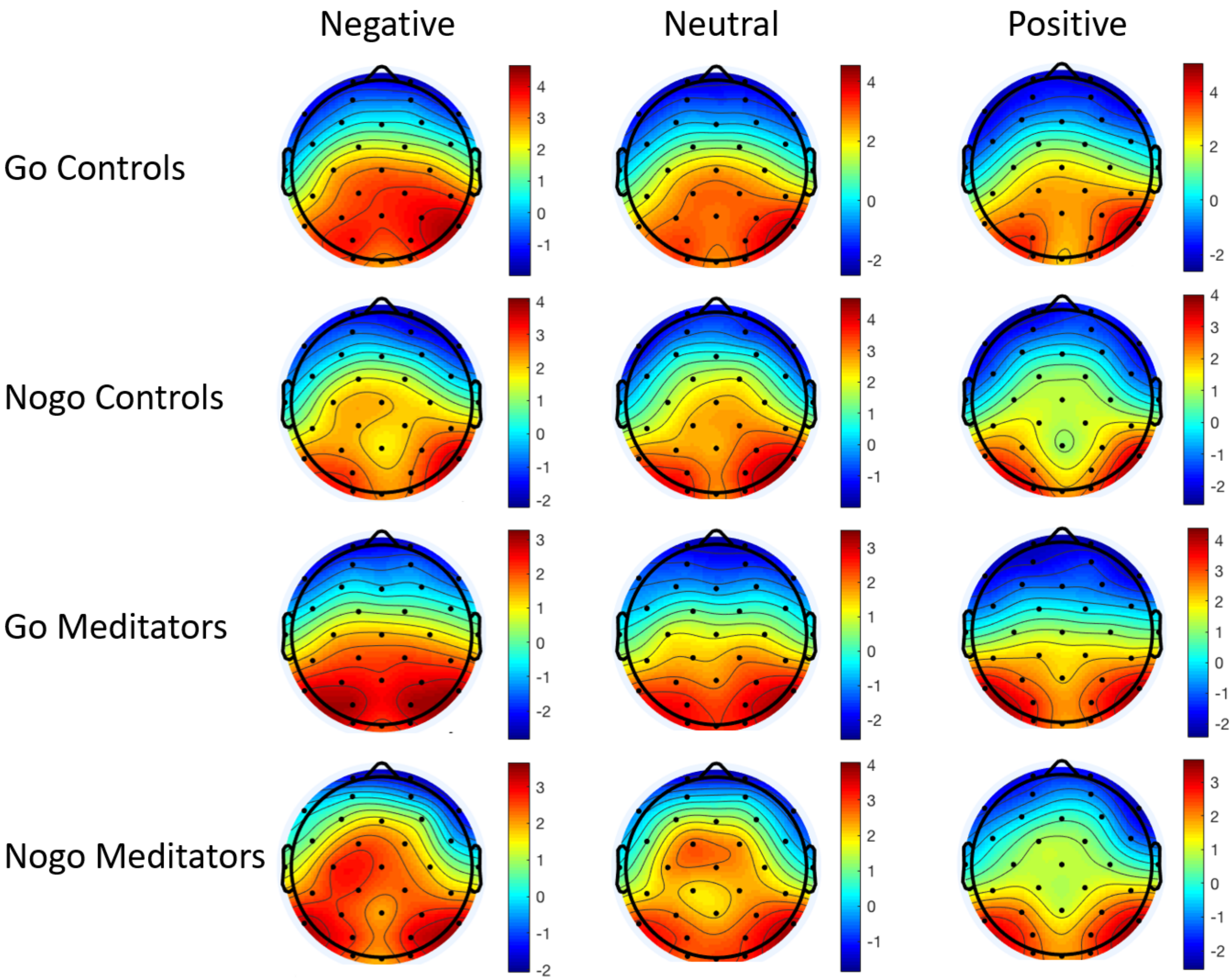
Negative
Neutral
Positive
Go Controls
Nogo Controls
Go Meditators
Nogo Meditators

Fig S2.
